# The challenge of chemotherapy-related cognitive impairment: assessing and managing cognitive decline after cancer treatment

**DOI:** 10.3332/ecancer.2025.1958

**Published:** 2025-08-05

**Authors:** Lucas Tadeu Barrak Stangler, Amanda Acioli de Almeida Robatto, Pedro José Galvão Freire, Gilberto de Castro Junior

**Affiliations:** 1 Instituto do Câncer do Estado de São Paulo, Universidade de São Paulo, São Paulo 05508-220, Brazil; 2 Hospital Sírio Libanês, São Paulo 01308-050, Brazil; 3 Rede D’Or de Oncologia, Recife 50070-170, Brazil; a https://orcid.org/0000-0002-8232-8733; b https://orcid.org/0000-0002-0255-0765; c https://orcid.org/0000-0002-3618-3609; d https://orcid.org/0000-0001-8765-3044

**Keywords:** chemobrain, chemotherapy-related cognitive impairment, cognitive function, chemotherapy

## Abstract

Chemotherapy-related cognitive impairment (CRCI) refers to a decline in cognitive function in patients during and after cancer treatment and is mainly associated with the use of cytotoxic chemotherapy (CT). As CT is still an essential component in the treatment of many cancers and taking into account the fact that cancer survival rates are increasing, CRCI may negatively impact the quality of life and working capacity of a growing number of individuals, especially those treated with curative intent in conditions such as breast cancer. There is still a need to address several issues related to CRCI, namely: the improved identification of risk factors, earlier diagnosis, more effective treatment strategies and prevention. Currently, the diagnosis relies on a multidisciplinary evaluation using neuropsychological tests, and rehabilitation remains the only treatment option available. A better understanding of the pathophysiological basis of CRCI is essential to improve the daily care and long-term outcomes of these patients.

## Introduction

Cognition encompasses the mental processes involved in the acquisition, storage, manipulation and processing of information, and is intimately linked to learning and the construction of knowledge through a set of cognitive abilities that include attention, memory, orientation, processing and executive functions, among others [1].

Cancer treatment has been shown to be a risk factor for cognitive decline [2]. This is mainly related to the treatments, with chemotherapy (CT) being strongly associated with cognitive complaints [3], although other treatment modalities such as radiotherapy, hormone therapy and immunotherapy may also be implicated. Oxidative damage, central nervous system invasion, genetic predisposition and aging can also be factors in this cognitive decline. Moreover, advances in the diagnosis and treatment of cancer have led to greater life expectancy among cancer patients, thus increasing the potential impact of cognitive decline associated with cancer treatment in respect of long-term quality of life in this population [4].

This condition is known as chemotherapy-related cognitive impairment (CRCI) or cancer treatment cognitive impairment and, when CT is the treatment, as chemotherapy-related cognitive dysfunction or more colloquially *‘chemobrain’* or *‘chemofog’* [2, 5, 6]. CRCI most commonly affects memory, executive functions, attention and information processing speed [7]. CRCI was first observed in long-term survivor patients, particularly those with breast cancer [8], but it has also been observed in other cancers. The accurate diagnosis of CRCI is crucial for its treatment, and it can significantly impact quality of life, affecting the individual’s ability to perform daily activities, work and maintain social relationships. Recognising CRCI allows healthcare providers to assess its severity and impact of cognitive impairment and tailor interventions accordingly.

## Epidemiology

The incidence of CRCI in the literature is estimated to be between 15% and 75% [3, 9, 10]. These divergent findings are probably due to the lack of a uniform definition in the literature, the difficulties in assessing cognition and the different tools being used for diagnosis across studies. Furthermore, it can be difficult to differentiate between CRCI and the cognitive decline that is associated with aging in the wider population.

In an online survey with 1,610 participants, with over 85% being breast cancer survivors, based on subjective self-reported data, it was found that 75% of patients presented cognitive complaints [3]. However, in studies analysing objective decline, a prevalence of 15%–25% [11] to 61% [9] was reported. In addition, in a recent meta-analysis of 52 studies of breast cancer survivors, the prevalence of CRCI was 44% using self-report tools, 16% using short cognitive screening tools and 21%–34% using neuropsychological test batteries [12].

While most CRCI research focuses on breast cancer, cognitive decline is not confined to this group. In a broader cohort of cancer survivors, including those with colorectal, lung and gynecological cancers, nearly 50% experienced new or worsening cognitive problems after treatment [13]. In colorectal cancer survivors, approximately 40% reported memory and executive-function difficulties regardless of chemotherapy exposure, suggesting that cognitive decline may be attributed to the cancer itself, rather than exclusively to treatment [14]. These findings highlight the multifactorial nature of CRCI, with contributions from both the disease and the treatment.

It is worth noting that a considerable number of patients had cognitive alterations prior to treatment, with about 35% of patients presenting persistent cognitive changes for months to years after treatment [10]. In relation to age, it was found that the level of worsening was similar in older adults and younger people, but as they start from different cognitive baselines, the burden may be greater in older adults. A study that evaluated patients with breast cancer treated in an adjuvant setting (*N* = 60 years and mean age 51.7 years) found that older patients with lower baseline cognitive reserve performed worse in post-CT processing speed tests than those not exposed to CT (*p* < 0.003) [4]. Thus, lower cognitive reserve is associated with potential vulnerability to a worse decline after treatment.

## CRCI mechanisms

The mechanisms underlying CRCI are not fully understood, although several theories exist, and it has been shown that the direct effects of cancer on cognition do not only come from tumour lesions in the central nervous system (CNS). Moreover, cancer can cause cognitive alterations even in patients without any neoplastic brain involvement (and before performing any cancer treatment). It is likely that these changes are due to the oxidative DNA damage in neural and glial cells found in cancer patients [4, 15].

Drug-induced damage has a role, possibly acting alongside some individual predisposition factors. These can include age or carrying an allele that increases the risk of dementia, such as the epsilon 4 allele of the Apolipoprotein E gene (APOE ε4) [16]. It is important to note that these mechanisms are not mutually exclusive, and multiple factors may contribute to the development of CRCI, which is very likely to be a multifactorial entity.

A recent review by Schagen *et al* [17] enumerated some of the main mechanisms involved in CRCI, namely synaptic dysfunction, impaired telomerase maintenance leading to DNA damage, microglial activation, neuronal stem cell dysfunction, impaired myelin integrity and production, cellular senescence, inflammation, disruptions to the blood–brain barrier (BBB), mitochondrial dysfunction, compromised oxidative and protein homeostasis and changes in the gastrointestinal microbiome. Other mechanisms also described included alterations in exosome cargo, and in the interaction between the CNS and peripheral nervous system [17].

In other recent studies, one of the dominant processes has been reported to be immune-mediated tissue damage, through a chronic inflammatory state. There is an increase in the inflammatory response during and after cancer treatment, mainly of pro-inflammatory cytokines, with macrophage and microglia activation, resulting in neuroinflammation, oxidative stress and neuronal loss, which ultimately is associated with cognitive impairment [2, 18].

As for specific drugs and their mechanisms of actions, several drugs have been implicated. These include cisplatin, which injures dendritic cells, and Adriamycin, which affects neuronal plasticity [19, 20]. In animal models, the combination of cyclophosphamide and Adriamycin, often used in breast cancer treatment, leads to inflammation and upregulated MAPK pathways resulting in oxidative stress damage to the nucleic acids of rat models hippocampus [20]. Adriamycin impairs the autophagy-lysosome system in mice neurons, which results in the accumulation of damaged material that manifests as neurotoxicity (Table 1) [21]. Adriamycin, carmustine, methotrexate and cyclophosphamide have been linked to oxidative stress [22], and in animal models, cisplatin has been implicated in gut dysbiosis leading to liver injury and oxidative stress that could affect the BBB [23]. These are just some examples of specific drugs that can cause damage and have been implicated in the development of CRCI (Table 1). Dosing, route of administration, the presence of structural brain lesions, prior or concurrent irradiation and interactions with other drugs can also influence the development of CRCI [24].

**Figure 1.**
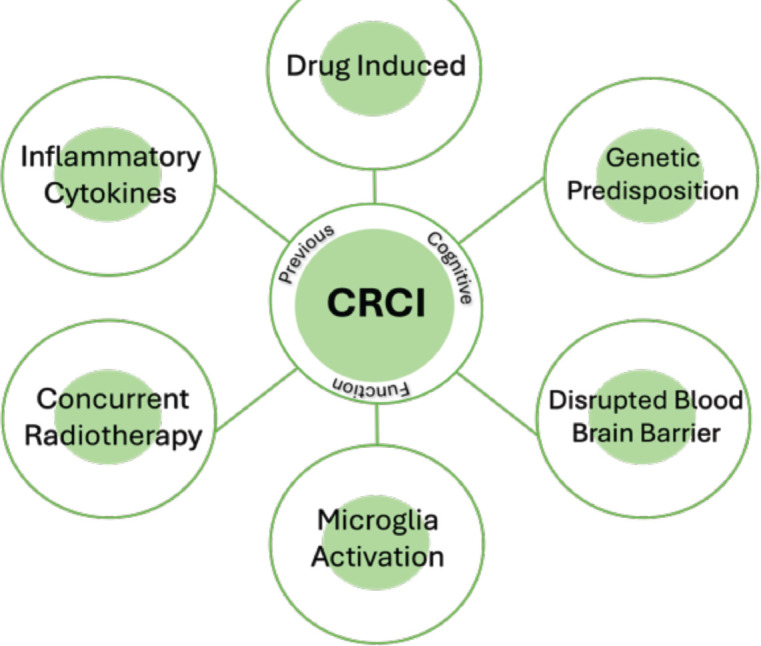
CRCI is a multifactorial disease.

CRCI results from a multifactorial interplay of neurotoxicity, inflammation, oxidative stress and genetic predisposition (Figure 1). Chemotherapy-induced damage interacts with pre-existing vulnerabilities, such as APOE ε4 or aging, to exacerbate cognitive decline. Further on, chronic inflammation disrupts the blood–brain barrier and impairs synaptic integrity, whereas mitochondrial dysfunction further compromises neuronal resilience. Even gut dysbiosis may contribute by perpetuating systemic oxidative stress and neuroinflammation. These mechanisms form a reinforcing cascade, where initial insults trigger secondary processes that worsen cognitive impairment [4]. Understanding these intricate interactions is crucial for developing interventions that target multiple pathways rather than addressing CRCI as a single-factor phenomenon.

### Mechanisms considered in this review [1]

Chemotherapy-related cognitive impairment results from a combination of neuroinflammation, oxidative stress and direct neurotoxic effects. Cytotoxic agents trigger the release of pro-inflammatory cytokines, activating microglia and disrupting the blood–brain barrier, which sustains a chronic inflammatory state. Concurrently, oxidative damage to neuronal mitochondria compromises energy production and synaptic function, leading to neuronal injury and cell death. These interconnected processes amplify one another in a multifactorial cascade, explaining why single‐target interventions often fall short and highlighting the need for comprehensive strategies to prevent and manage CRCI.

## Diagnosis

Usually, the triggers for the investigation of CRCI are complaints such as concentration problems and difficulties in remembering names and numbers, word finding or multitasking [26].

In order to diagnose CRCI it is important to have an initial evaluation, understanding if symptoms were present previously to CT, as well as whether other symptoms such as depression, anxiety, pain, fatigue or insomnia are present. In addition, it might be helpful to run blood tests and have image scans to exclude other causes of cognitive decline such as dementia, hypothyroidism, vitamin deficiencies, chronic infections and cardiopulmonary impairments, among other factors.

There are several tests that can be applied to detect cognitive dysfunction during CT. An effort is being made to try to standardise the diagnosis, since there is heterogeneity in the tests used to assess CRCI in different studies. The International Cancer and Cognition Task Force (ICCTF) recommends the use of neuropsychological tests that assess the most objective impaired cognitive domains [27] (Table 2).

In order to test learning and memory, processing speed and executive function, the ICCTF recommends the Hopkins Verbal Learning Test-Revised (HVLT-R) [28], the Trail Making Test [29] and the Controlled Oral Word Association test of the Multilingual Aphasia Examination [30]. Cognitive decline is established when patients have scores at or below −1.5 SDs from the normative mean (or from a control group) in two or more of the previously mentioned tests or −2.0 SDs in one test.

However, differentiating between CRCI and neurodegenerative disease can be difficult in clinical practice, and having an evaluation performed by a neuropsychologist is sometimes not feasible. The Montreal Cognitive Assessment [35] does not require a neuropsychologist and works as a screening test. If the test indicates the presence of any cognitive impairment, the patient should be referred for a neuropsychological assessment [36].

Another helpful tool for assessing cognitive difficulties in cancer survivors is the Functional Assessment of Cancer Therapy-Cognition (FACT-Cog) *version 3* [37] which is a self-reported 37-item questionnaire that evaluates the physical, social, emotional and functional well-being domains. FACT-Cog consists of four subscales: Perceived cognitive impairment-CogPCI (20 items), Perceived Cognitive Ability-CogPCA (9 items), Comments from Others on Cognitive Function-CogOth (4 items) and Impact on Quality of Life-CogQoL (4 items). The total score for the FACT-Cog is computed by summing all the item scores and ranges from 0 to 148 points, with a higher score indicative of better perceived cognitive functioning. Studies describing cut-off scores are scarce in literature; however, in an analysis of 133 breast cancer survivors a cutoff score below 54, with 76% sensitivity and 82% specificity, was identified for the 18-item perceived cognitive impairment (PCI) subscale, and a cutoff score below 60, with 76% sensitivity and 84% specificity, for the 20-item PCI [38].

As for diagnosis, imaging tests have shown anatomical and functional changes in the central nervous system after chemotherapy. Changes in gray matter volume and density, reductions in white matter microstructure and alterations in brain activity and connectivity have been described, being associated with worse performance in neuropsychological tests. In contrast, these studies also demonstrated areas of hyperactivation and hyperconnectivity, which can be interpreted as compensatory mechanisms [2, 39]. Magnetic resonance imaging (MRI) can be helpful in the diagnosis of CRCI, especially if combined with clinical findings and cognitive tests, with neuroimaging data showing a reduction in gray matter density in cancer patients in frontal, parietal and temporal regions [40].

Recent advances in neuroimaging, particularly functional MRI (fMRI), have provided deeper insights into the neural correlates of CRCI. Unlike purely structural studies, fMRI evaluates brain activation during cognitive tasks, revealing dynamic patterns of dysfunction. In a comprehensive systematic review, Simó *et al* [41] demonstrated consistent alterations in both structural and functional imaging studies, with reduced activation in frontoparietal networks involved in executive function, attention and working memory in cancer survivors treated with chemotherapy. Interestingly, some of these alterations were also observed in patients who did not receive chemotherapy, suggesting that both cancer itself and its treatments contribute to brain functional changes. These findings highlight that CRCI reflects a complex disruption of brain networks and may inform future studies employing functional imaging to further explore cognitive changes in cancer survivors [41].

Thus, although there is a significant difficulty in diagnosing CRCI in clinical practice, paying attention to the patient’s clinical history – especially if there was any cognitive complaint prior to CT, excluding other causes of cognitive decline, and having a multidisciplinary approach that includes a neuropsychologist can lead to a more precise diagnosis.

## Biomarkers

To identify cancer patients at greater risk of developing cognitive decline or with already established cognitive impairment, various studies have focused on the attempt to identify diagnostic biomarkers. In a review published by Országhová *et al* [4], biomarkers were divided into four categories: genetic, plasma, cerebrospinal fluid (CSF) and radiological. While no specific biomarkers have been definitively established for CRCI, several potential markers have been investigated in research studies. The following biomarkers have been explored:

**Inflammatory markers**: Cytokines (e.g., interleukin-6, tumour necrosis factor-alpha and interleukin-1B) [42, 43] have been associated with inflammation and cognitive dysfunction in breast cancer patients in small studies. Another study evaluated 400 breast cancer survivors and found an association between chronic inflammation expressed through high C-reactive protein levels and cognitive decline [44].

**Brain imaging biomarkers**: Functional and structural brain imaging techniques, such as MRI and positron emission tomography (PET), have been used to identify brain changes associated with CRCI. Li and Caeyenberghs [40] summarised in a review possible alterations found in MRI, such as a reduction of gray matter density in cancer patients in frontal, parietal and temporal regions, with a moderate-to-strong correlation between worse cognitive function and morphological changes in frontal brain regions. In addition, changes in brain function (brain activation and cerebral blood flow) involving frontal, parietal, occipital, temporal and cerebellar regions have been described. In diffusion-weighted MRI, it has been suggested that a reduction in white matter integrity involving the superior longitudinal fasciculus, corpus callosum, forceps major and corona radiata and altered structural connectivity across the whole brain network can be markers of cognitive impairment. Thus, neuroimaging tests could bring additional information to corroborate the association between the observed cognitive decline and cancer treatment. Fluorodeoxyglucose PET scans can also be helpful. In a study with 21 participants treated for non-Hodgkin Lymphoma a significant reduction in brain metabolism or ^18^FDG uptake was found in all regions of the brain, but particularly in the mesial temporal and frontal lobes after chemotherapy [45].

**Genetic markers**: Associations between genetic polymorphisms and the development of cognitive decline have been evaluated, mainly involving genes associated with neurogenesis, repair and neuroplasticity after neuronal damage such as *APOE* ε4 and Brain-Derived Neurotrophic Factor *(BDNF).* In a study by Ahles *et al* [46], long-term survivors of breast cancer (mean 8.8+/−4.3 years after treatment) with at least one *APOE* ε4 allele scored significantly lower in visual memory (*p* < 0.03) and spatial ability (*p* < 0.05) [46]. In addition, carriers of the *Met* allele of the *BDNF* gene experienced less impairment in the domains of verbal fluency and multitasking ability in comparison with those with the *Val/Val* homozygotes [47].

Another gene involved in catechol-O-methyltransferase (*COMT);* increased *COMT* activity is associated with the degradation of catecholamines, which results in higher availability of dopamine at the level of the prefrontal cortex leading to cognitive impact. In breast cancer patients, *COMT-Val* carriers had worse performance in tests of attention, verbal fluency and motor speed [48]. In addition, the *rs165599* polymorphism in the *COMT* gene was associated with impaired retrospective memory [49].

Overexpression of some miRNAs such as miRNA-206, miRNA-132 and miRNA-134 could be a biomarker of early cognitive decline, as some studies have associated them with mild cognitive impairment in Alzheimer’s disease [50–52]. These miRNAs target BDNF and SIRT1, which are involved in cognition, and although they were not evaluated in association with CT, this is an area that could be further explored [2].

The review by Országhová *et al* (2021) [4] highlights some other potential biomarkers such as exosomes, which are small endocytic vesicles that could have a role in the development of CRCI [53], as well as the gut microbiome which could influence brain function via the production of short-chain fatty acids [54].

It is important to note that research on biomarkers for CRCI is still evolving, and further investigations are needed to validate and establish these potential markers. Additionally, individual variations in treatment regimens, cancer types and patient characteristics may influence the presence and significance of specific biomarkers in CRCI.

The identification of new biomarkers is essential for detecting early CRCI onset or identifying individuals at risk of developing CRCI. This may enable clinicians to develop and apply early therapeutic interventions, potentially mitigating the long-term impact of cognitive changes on quality of life. However, much work is still needed to translate these findings into clinical applications, as studies on these biomarkers are still primarily conducted in animal models. For example, in a recent study conducted by Usmani *et al* [55], increasing BDNF levels with riluzole in animal models has been shown to prevent chemotherapy-induced reductions in hippocampal BDNF levels, leading to significant improvements in hippocampal-dependent learning and memory function (spatial recognition), fear extinction memory consolidation and reduced anxiety-like behaviour.

In summary, although several biomarkers are being studied, there are limitations in their practical application as most are still under investigation, and there is a lack of validation from large-scale studies. Furthermore, as CRCI is a multifactorial disease, it is likely that more than one biomarker is involved.

## Treatment

### Pharmacological interventions

The management of CRCI includes both pharmacological and non-pharmacological approaches. While several pharmacological treatments have been explored, the results remain inconsistent and their benefits are limited in many cases. These treatments include neurostimulants (e.g., methylphenidate and modafinil), anti-dementia drugs (e.g., donepezil and memantine) and other agents such as Ginkgo biloba, erythropoietin and antioxidants [36]. Despite the exploration of these options, no pharmacological intervention has been universally recommended for CRCI, highlighting the need for further large-scale clinical trials to elucidate the mechanisms and potential benefits of these treatments.

Existing clinical trials are often limited by heterogeneity in patient populations, such as variations in baseline cognitive impairment, prior cancer treatments, comorbidities and medications that may influence brain function and recovery [36, 56]. Furthermore, most studies have been open-label or single-arm trials, which are susceptible to biases, such as the placebo effect or improvements driven by repeated cognitive testing, rather than genuine therapeutic effects. These factors contribute to conflicting results, making it challenging to determine the true efficacy of pharmacological treatments for CRCI.

For example, donepezil, a cholinesterase inhibitor, has been studied for its potential to treat CRCI, with promising results in both preclinical and clinical studies. In a preclinical study, Winocur *et al* [61] demonstrated that donepezil improved hippocampal-dependent memory, including spatial memory, in animal models [61]. Building on these findings, a clinical trial by Lawrence *et al* (2016) [92] administered donepezil for 24 weeks to breast cancer survivors post-chemotherapy. This group showed significant improvements in memory compared to a placebo group (Lawrence *et al*, 2016). However, other studies have shown mixed results, with some reporting no significant improvements in cognitive function [57]. These inconsistencies highlight the complexity of pharmacological treatment for CRCI, with factors such as chemotherapy regimens, baseline cognitive function and patient characteristics likely contributing to these divergent outcomes.

Memantine, an N-methyl D-Aspartate (NMDA) receptor antagonist, has been explored for its ability to reduce radiation-induced neuronal damage in brain tumour patients. This suggests that memantine could help prevent cognitive decline induced by radiation therapy. However, its efficacy for CRCI in non-CNS cancer survivors, particularly those without brain metastasis, remains unclear. Further studies are necessary to assess the role of memantine in CRCI treatment and to explore its broader application in various cancer populations [57].

In addition to these treatments, novel pharmacological agents are being explored in ongoing trials. These include neurostimulating, neuroprotective and anti-neuroinflammatory agents. Early animal studies suggest that substances like the antidepressant fluoxetine, cotinine (a nicotine derivative) and the antioxidant zinc sulfate may improve cognitive performance following chemotherapy. However, further clinical trials are needed to determine their efficacy in cancer survivors [4, 56, 57].

### Non-pharmacological interventions

Regarding the non-pharmacological strategies, most of them focus on rehabilitation. Trying to rehabilitate the patient is of the utmost importance. Rehabilitation from CRCI involves a multidimensional approach that focuses on cognitive rehabilitation, lifestyle adjustments and supportive care. Even though there is no cure for CRCI, various strategies and interventions can help manage the cognitive changes and improve overall cognitive function. Below, some potentially useful strategies with clinical relevance are described.

### Cognitive rehabilitation exercises

Cognitive rehabilitation exercises typically involve computerised cognitive training programs targeting domains such as attention, processing speed, memory and executive function. These programs deliver structured, repetitive tasks aimed at improving specific cognitive abilities through adaptive learning. Objective neuropsychological measures have shown that computer-based cognitive training can lead to significant improvements, particularly in processing speed and working memory. Such interventions are increasingly accessible through home-based platforms and have been associated with durable cognitive benefits in cancer survivors [2, 4].

### Psychoeducational and compensatory strategy skills

Psychoeducational and compensatory interventions focus on helping patients manage cognitive challenges in daily life through practical strategies. Memory and Attention Adaptation Training (MAAT), a cognitive-behavioural intervention, teaches techniques such as the use of external memory aids, stress reduction and pacing strategies to minimise cognitive overload. In addition to initial findings, a brief, group-based version of MAAT demonstrated feasibility and effectiveness in breast cancer survivors, showing improvements in cognitive complaints and self-reported functioning [58]. Goal Management Training, originally developed for individuals with brain injuries, has also shown efficacy in cancer populations, improving executive function and daily task performance in brain tumour survivors [59]. These low-intensity interventions are appealing for clinical environments where access to specialised cognitive rehabilitation services may be limited.

### Physical exercise

Although physical exercise seems promising in animal models [60, 61], in humans there is still a need to address the optimal timing, duration, mode or intensity of the exercise in the context of CRCI treatment and/or prophylaxis [2], with a lack of strong robust data from meta-analysis regarding this topic. In a systematic review of 29 trials, Campbell *et al* [62] found that the evidence supporting exercise as a strategy to address CRCI is limited, as further research and better endpoints are needed to confirm the possible role of exercise in preventing and managing cognitive impairments [62].

### Mindfulness and neurofeedback

Mindfulness-based interventions, including yoga and tai chi, are gaining attention as promising strategies for addressing CRCI. These mind-body practices cultivate sustained present-moment awareness and non-judgmental attention, processes that not only alleviate emotional symptoms such as anxiety and depression but also directly ‘exercise’ attentional and executive control systems, which are often hypoactive following chemotherapy. A possible explanation for their effectiveness is that the practice of becoming more aware of thoughts and feelings, and relating to them as transient mental events rather than emotional triggers, reduces psychological distress and enhances cognitive flexibility [63, 64].

Yoga has been shown to be feasible and safe when delivered remotely or in group formats, offering an accessible and low-risk intervention compared to other physical activities that may require supervision to avoid injury. In breast cancer survivors, yoga interventions have been associated with significant improvements in self-reported cognitive function, particularly in domains such as memory and attention [65]. Similarly, tai chi has demonstrated benefits for cognitive complaints and overall quality of life among cancer survivors [66]. Although evidence remains preliminary, these approaches offer promising, low-intensity options for managing CRCI in survivorship care.

Neurofeedback also had an impact on improving cognition in breast cancer survivors in a small study with 23 participants [67].

### Sleep optimisation

Insomnia affects 60% of cancer patients, and some studies found a relationship between cognitive decline and sleep disturbances [68]. In patients with Alzheimer’s disease, treating sleep disturbances improved cognitive function [69]. Strategies used to improve the quality of sleep include behavioural changes and pharmacological interventions. Other evaluated strategies associated with benefits were treatment with melatonin [70] and cannabidiol [71]; however, studies have only involved a small number of cancer patients.

#### Other causes of cognition impairment in cancer survivors

Although this review focuses primarily on CRCI, cognitive dysfunction in cancer survivors can arise from multiple sources, including the cancer itself, systemic inflammation and other treatment modalities beyond chemotherapy [2, 4, 10]. While CRCI is most strongly associated with exposure to cytotoxic agents, it is increasingly recognised that baseline cognitive changes may predate treatment initiation in some patients [4, 15]. For clarity, we briefly summarise non-chemotherapy-related cognitive impairments below, maintaining the primary focus of this review on CRCI.

### Radiotherapy

Radiotherapy (RT) can cause cognitive decline through its action on mediating glial cell activation and the increase in glutamatergic neurons, which leads to excitotoxicity and ultimately cellular death. The main affected domains are attention, executive functions, processing, learning and memory. Cognitive decline after radiotherapy may appear soon after the treatment, or months or years following the exposure. The former is more likely to be restored over time, but the latter is more likely to remain the same or worsen [2, 18].

An important strategy to mitigate the cognitive side effects of brain irradiation is to spare eloquent areas, such as the hippocampus, whenever possible [72]. Stereotactic radiosurgery, another radiotherapy technique, can spare brain tissue and preserve cognition [73]. Also, the use of memantine, an NMDA receptor antagonist, during RT was shown to be neuroprotective, in the phase III trial RTOG 0614 the association with whole brain radiotherapy (WBRT), produced a relative reduction in the rate of cognitive dysfunction of 22% [74]. In the NRG CC001 study, memantine was evaluated in association with hippocampal avoidance WBRT (HA-WBRT), and the risk of cognitive failure was lower after HA-WBRT plus memantine versus WBRT plus memantine (adjusted hazard ratio, 0.74; 95% CI, 0.58–0.95; *p* = 0.02) [75].

### Endocrine therapy

Endocrine therapy such as androgen deprivation therapy, the use of aromatase inhibitors and antiestrogens are linked to endocrine disorders in the hypothalamo–pituitary–adrenal axis. In a 6-year follow-up of breast cancer patients receiving hormone therapy, no detrimental effect on objective measures was found; however, there were more subjective cognitive complaints in patients receiving hormone therapies. In prostate cancer patients, little effect on cognition was found (effect size, *g* = −0.67) with visuomotor functions being the most impaired domain. Another interesting result was that patients taking enzalutamide were more likely to have cognitive complaints than patients taking acetate abiraterone and prednisone [39].

### Targeted therapies

Target therapies are also associated with cognitive complaints, possibly related to reduced angiogenesis and reduced cerebral blood flow impacting neurogenesis. Although many Vascular Endothelial Growth Factor (VEGF) inhibitors do not cross the BBB, the possible explanation for their impact on cognition is through inhibition of peripheral VEGF [76].

Studies evaluating anti-VEGF showed that 31% of patients with metastatic renal carcinoma presented cognitive decline [77]. In patients with metastatic renal carcinoma and gastrointestinal stromal tumour being treated with sunitinib or sorafenib, there was a worse performance in neurocognitive tests with a more significant impact on executive functions, learning and memory [78].

Lastly, in the CROWN trial, lorlatinib was also associated with cognitive decline in 21% of patients. This third-generation ALK inhibitor has also been reported to cause mood disorders and suicide ideations [79], probably due to its high penetrations in CNS.

### Immune checkpoint inhibitors

Checkpoint inhibitors can cause immune-mediated neurotoxicity, such as demyelinating encephalitis and autoimmune encephalitis [80]. Although rare, with an incidence of 0.1%, autoimmune encephalitis has the potential to lead to a rapidly progressive dementia syndrome. In small unicentric studies, a cognitive impact was found in 32% and 41% of patients treated with pembrolizumab and ipilimumab for melanoma, respectively [81, 82].

### T-cell therapy

Cytokine release syndrome and immune effector cell-associated neurotoxicity syndrome, potential complications of T-cell therapies, can have an impact on cognition. Belin *et al* [83] found that cognitive impairment could be severe in up to 36% of patients treated with CAR-T-cell therapy for diffuse large B cell lymphoma [83], with the most affected domains being executive functioning, memory and attention [84]. One possible mechanism of the damage associated with T-cell therapy is the apoptosis caused by neuroinflammation that is induced through cytokine release [20, 85].

### Psychological and psychiatric causes

Psychiatric diseases such as depression are common after cancer diagnosis and treatment, and depression could be a cause of cognitive impairment, as it causes structural and functional disturbances in neural circuits [86]. In a study with 136 breast cancer patients, depression had a significant partial mediating effect between objective cognitive functioning and QoL (Z = 2.62, *p* = 0.009) analysed through the Sobel test [87]. Another cross sectional with 5,078 patients found that moderate to severe cognitive symptoms could be associated with depression (OR 1.92; 95% CI, 1.59–2.31) and anxiety (OR 1.57; 95% CI, 1.30–1.89) [88].

In summary, cognitive impairment in cancer survivors reflects a spectrum of disease- and treatment-related processes [2, 10]. CRCI arises from a convergence of neuroinflammation, oxidative stress, mitochondrial dysfunction and direct neurotoxic effects [2, 5, 18]. These pathways frequently intersect with mechanisms activated by other therapies, such as radiotherapy and immunotherapy, underscoring the multifactorial and overlapping nature of cognitive changes in this population.

## Future perspectives

Given the increasing number of cancer survivors, there is an urgent need to reduce the long-term symptoms that can impact the quality of life and working capacity of these patients. The future of the treatment of CRCI lies in a better understanding of the molecular mechanisms involved, which could help in the diagnosis and the identification of potential targets for treatment.

A better understanding of the mechanisms that lead to neuroinflammation and potential targets could also help to address CRCI more effectively. Some studies are underway to try to elucidate the mechanisms related to CRCI, such as the BioCAN (NCT05280262) which will evaluate potential CSF biomarkers for cognitive impairment in children undergoing treatment for acute lymphoblastic leukemia or lymphoblastic lymphoma, and NCT05014399 is evaluating plasma biomarkers in patients receiving chemotherapy for colorectal cancer. In addition, there are several studies investigating interventions that could mitigate CRCI such as the NCT06508671 trial, which is investigating the role of DL-3-n-butylphthalide as a CRCI prophylactic agent. DL-3-n-butylphthalide showed improvement in cognitive and global functioning in patients with vascular dementia by inflammatory response and other mechanisms [89]. As inflammation plays a pivotal role in CRCI development this trial hypothesised that DL-3-n-butylphthalide could also work on patients with CRCI. In NCT06686823, the impact of a training program on cognition during chemotherapy for breast cancer (NCT04789187) is under evaluation.

## Conclusion

Cognitive complaints, especially in memory and processing speed, are common among cancer survivors. Although patients will often experience an improvement in symptoms 6 months after CT completion [90], some studies report 46%–60% of patients with persistent symptoms [13, 91]. Cognitive changes can significantly impact the quality of life, affecting the ability to perform daily activities, to work and to keep up social relationships. The diagnosis and identification of CRCI is important, as it allows healthcare providers to assess the severity and impact of the cognitive impairment and tailor interventions accordingly. However, the recognition of CRCI can be difficult, and a comprehensive neuropsychological evaluation, especially in environments with limited resources, is not always feasible. Beyond the diagnosis, another challenge is the treatment of the affected patients, as pharmacological treatment for CRCI has limited efficacy, although rehabilitation strategies seem to improve patients' cognition, and should be encouraged.

It is now clear that cancer and its treatment can impact different brain areas, and CRCI is a real and significant phenomenon, affecting memory, concentration and overall cognitive functions. Its exact mechanisms are complex and multifactorial, and not yet fully understood although the current evidence indicates that they involve the direct neurotoxic effects of chemotherapy, inflammatory responses and possible genetic predispositions. Recognising CRCI is crucial for validating patients' experiences and beginning rehabilitation as soon as possible, as this is the only intervention that has so far been shown to have a significant impact on improving the patients' quality of life. Further multidisciplinary research is urgently required to help deepen our understanding of this condition and develop strategies to mitigate its impact on cancer survivors by developing more efficient preventative measures and targeted interventions.

## List of abbreviations

ALL, Acute lymphoblastic leukemia; APOE ε4, epsilon 4 allele of the Apolipoprotein E gene; BDNA, Brain-Derived Neurotrophic Factor; BBB, Blood–brain barrier; CBD, Cannabidiol; CBT, Cognitive behavioural therapy; CNS, Central nervous system; COMT, Catechol-O-methyltransferase; CRCD, Chemotherapy-related cognitive dysfunction; CRCI, Chemotherapy-related cognitive impairment; CRP, C-reactive protein; CRS, Cytokine release syndrome; CTCI, Cancer treatment cognitive impairment; CSF, Cerebrospinal fluid; FACT-Cog, Functional Assessment of Cancer Therapy-Cognition; FDG-Pet, Fluorodeoxyglucose PET scans; Fmri, Functional magnetic resonance imaging; GIST, Gastrointestinal stromal tumour; HA-WBRT, Hippocampal avoidance whole-brain radiotherapy; HVLT-R, Hopkins Verbal Learning Test-Revised; ICANS, Immune effector cell-associated neurotoxicity syndrome; ICCTF, International Cancer and Cognition Task Force; LBL, Lymphoblastic lymphoma; MAAT, Memory and Attention Adaptation Training; MBI, Mindfulness-based interventions; MRI, Magnetic resonance imaging; NMDA, N-methyl D-Aspartate; OR, Odds Ratio; PET, Positron emission tomography; RCT, Randomised controlled trial; RT, Radiotherapy; SRS, Stereotactic radiosurgery; SCFAs, Short-chain fatty acids; TMT, Trail Making Test; VEGF, Vascular Endothelial Growth Factor; WBRT, Whole brain radiotherapy.

## Conflicts of interest

The authors declare no conflicts of interest in respect of this work.

## Author contributions

Conceptualisation and design: Gilberto de Castro Junior.

Manuscript writing: Amanda Acioli de Almeida Robatto, Lucas Tadeu Barrak Stangler, Pedro José Galvão Freire.

Final approval of manuscript: All authors.

Accountable for all aspects of the work: All authors.

## Figures and Tables

**Table 1.** Possible mechanisms involved in CRCI [4, 3, 25].

Chemotherapy agent (brand name)	Affected domain^1^	Possible mechanisms	Use				
Doxorubicin (Conventional – Adriamycin^®^, Rubex^®^) (Pegylated liposomal – Caelyx^®^, Doxil^®^, Lipodox^®^)	Clinical studies: executive functions, language, memory, processing speed Animal models: short term memory	Inflammation Oxidative stress Mitochondrial dysfunction Apoptosis induction Decreased neurogenesis Synaptic changes and downregulation of neurotransmitters	Acute lymphoblastic leukemia*, Adrenocortical carcinoma*, Adult T-cell leukemia/lymphoma*, Bladder carcinoma*, Breast cancer*, Endometrial cancer**, Ewing’s sarcoma*, Gastric cancer*, Hepatic carcinoma**, Hodgkin's Lymphoma*, Kaposi’s sarcoma***, Multiple myeloma*, Non-Hodgkin's Lymphoma*, Osteosarcoma*, Soft tissue sarcoma**, Thyroid carcinoma***, Thymoma*, Wilm’s tumour*				
Taxanes (Cabazitaxel – Jevtana^®^) (Docetaxel – Docefrez^®^, Taxotere^®^) (Paclitaxel – Taxol^®,^ Onxol^®^)	Clinical studies: attention, concentration, executive functions Animal models: spatial memory	Decreased hippocampal neurogenesis Changes in neuronal morphology	Cabazitaxel Prostate cancer***	Docetaxel Breast cancer**, Ewing's sarcoma*, Head and neck cancer*, Non-small cell lung cancer***, Osteosarcoma*, Ovarian cancer*, Prostate cancer**		Paclitaxel Bladder cancer**, Breast cancer**, Cervical cancer*, Endometrial cancer*, Esophageal cancer**, Head and Neck cancer*, Kaposi Sarcoma***, Non-small cell lung cancer*, Ovarian cancer**, Small cell lung cancer***	
Methotrexate (Jymlavo^®^, Otrexup^®^, RеdiΤrеx DSC^®^)	Clinical studies: association with leukoencephalopathy Animal models: visual and spatial memory, executive functions	Inflammation Microglia activation Damage to oligodendrocytes and impaired myelination Hippocampal neurogenesis suppression	Acute lymphoblastic leukemia**, Acute lymphocytic leukemia*, Acute promyelocytic leukemia***, Bladder cancer*, Breast cancer*, Burkitt lymphoma*, Choriocarcinoma**, Cutaneous T-cell lymphoma**, Head and neck cancer***, Leptomeningeal cancer***, Non-Hodgkin lymphoma*, Osteosarcoma*, Soft tissue sarcoma*				
Fluorouracil (Adrucil^®^, Efudex cream^®^, Fluorouracil injection)	Clinical studies: memory, processing, executive functions Animal Studies: spatial memory	Inflammation Mitochondrial dysfunction Damage to oligodendrocytes and impaired myelination Decreased neurogenesis	Basal cell skin cancer (topical)***, Bladder cancer*, Bowen's disease skin cancer (topical)***, Breast cancer*, Cervical cancer*, Colorectal cancer**, Esophageal cancer*, Gastric cancer*, Head and neck cancer*, Ocular cancer (topical)***, Pancreatic cancer*, Squamous cell skin cancer (topical)***				
Platinum-based (Carboplatin^®^ – Paraplatin) (Cisplatin^®^ – Platinol) (Oxaliplatin^®^ – Eloxatin)	Clinical studies: memory, learning, global cognitive decline Animal models: short- and long- term memory, executive functions	Inflammation Oxidative stress Mitochondrial dysfunction Damage to oligodendrocytes and impaired myelination Loss of microtubule stabilization	Carboplatin Anal cancer*, Bladder cancer*, Breast cancer**, Cervical cancer**, Endometrial cancer*, Esophageal cancer*, Ewing’s sarcoma*, Gastric cancer*, Germ cell tumour*, Head and neck cancer*, Hodgkin Lymphoma*, Malignant mesothelioma*, Melanoma*, Merkel cell carcinoma**, Neuroendocrine tumours*, Non-Hodgkin Lymphoma*, Non-small cell lung cancer*, Ovarian cancer*, Osteosarcoma*, Prostate cancer*, Small cell lung cancer*, Testicular cancer*, Thymoma*, Thyroid malignancies*		Cisplatin Adrenalcortical cancer*, Anal cancer*, Bladder cancer*, Breast cancer**, Cervical cancer**, CNS Lymphoma*, Endometrial cancer*, Esophageal cancer*, Germ cell tumour*, Gestational trophoblastic neoplasia*, Head and neck cancer**, Hodgkin lymphoma*, Mesothelioma*, Multiple myeloma*, Nasopharyngeal cancer*, Neuroendocrine tumours*, Non-Hodgkin lymphoma*, Non-small cell lung cancer*, Osteosarcoma*, Ovarian cancer*, Pancreatic cancer*, Penile cancer*, Prostate cancer*, Salivary gland cancer**, Small cell lung cancer*, Thymoma*, Urothelial cancer*		Oxalipatin Colorectal cancer*

^1^ summary of preclinical and clinical data * in combination with other chemotherapy agents; ** in combination or as single agent; *** single agent. Note: While certain chemotherapy agents are indicated for specific cancers, their use may be extended to others either in combination or as single agent, subject to regional approvals.

**Table 2.** International Cancer Cognition Task Force recommended tests [28].

Tests	Domains	Test duration
Mains tests		
HVLT-R [29]	Verbal memory and delayed recall	15–20 minutes for active administration + 20–25 minutes delay (for delayed recall)
Controlled Oral Word Association Test [31]	Speeded lexical fluency and executive function	5–10 minutes
Trail making test [30]	Psychomotor speed and executive function	5–8 minutes
Additional tests		
Auditory Consonant Trigrams [32]	Working memory, executive function, complex attention	10–15 minutes
WAIS-III letter-number sequencing [33]		5–7 minutes
Paced Auditory Serial Addition Test [34]		5–8 minutes
Brief test of attention [35]		5–10 minutes

## References

[1] de Alvarenga PG, Andrade AG (2008). Fundamentos em psiquiatria.

[2] Ahles TA, Root JC, Ryan EL (2012). Cancer- and cancer treatment-associated cognitive change: an update on the state of the science. J Clin Oncol.

[3] Lange M, Licaj I, Clarisse B (2019). Cognitive complaints in cancer survivors and expectations for support: results from a web-based survey. Cancer Med.

[4] Országhová Z, Mego M, Chovanec M (2021). Long-term cognitive dysfunction in cancer survivors. Front Mol Biosci.

[5] Mounier NM, Abdel-Maged AE, Wahdan SA (2020). Chemotherapy-induced cognitive impairment (CICI): an overview of etiology and pathogenesis. Life Sci.

[6] Ahles TA (2000). Chemo brain: it’s not in your head. In: 42nd Annual Science Writer’s Seminar.

[7] Joly F, Giffard B, Rigal O (2015). Impact of cancer and its treatments on cognitive function: advances in research from the Paris International Cognition and Cancer Task Force Symposium and update since 2012. J Pain Symptom Manag.

[8] Ahles TA, Saykin AJ, Furstenberg CT (2002). Neuropsychologic impact of standard-dose systemic chemotherapy in long-term survivors of breast cancer and lymphoma. J Clin Oncol.

[9] Wefel JS, Lenzi R, Theriault RL (2004). The cognitive sequelae of standard-dose adjuvant chemotherapy in women with breast carcinoma: results of a prospective, randomized, longitudinal trial. Cancer.

[10] Janelsins MC, Kesler SR, Ahles TA (2014). Prevalence, mechanisms, and management of cancer-related cognitive impairment. Int Rev Psychiatry.

[11] Ahles TA, Schagen S, Vardy J (2012). Neurocognitive effects of anti-cancer treatments. Clin Psycho-Oncol.

[12] Whittaker AL, George RP, O'Malley L (2022). Prevalence of cognitive impairment following chemotherapy treatment for breast cancer: a systematic review and meta-analysis. Sci Rep.

[13] Schmidt JE, Beckjord E, Bovbjerg DH (2016). Prevalence of perceived cognitive dysfunction in survivors of a wide range of cancers: results from the 2010 LIVESTRONG survey. J Cancer Surviv.

[14] Vardy JL, Dhillon HM, Pond GR (2015). Cognitive function in patients with colorectal cancer who do and do not receive chemotherapy: a prospective, longitudinal, controlled study. J Clin Oncol.

[15] Lange M, Hardy-Léger I, Licaj I (2020). Cognitive impairment in patients with breast cancer before surgery: results from a CANTO cohort subgroup. Cancer Epidemiol Biomarkers Prev.

[16] Fernandez HR, Varma A, Flowers SA (2020). Cancer chemotherapy related cognitive impairment and the impact of the Alzheimer's disease risk factor APOE. Cancers.

[17] Schagen SB, Tsvetkov AS, Compter A (2022). Cognitive adverse effects of chemotherapy and immunotherapy: are interventions within reach?. Nat Rev Neurol.

[18] Fleming B, Edison P, Kenny L (2023). Cognitive impairment after cancer treatment: mechanisms, clinical characterization, and management. BMJ.

[19] Andres AL, Gong X, Di K (2014). Low-doses of cisplatin injure hippocampal synapses: a mechanism for ‘chemo’ brain?. Exp Neurol.

[20] Liu RY, Zhang Y, Coughlin BL (2014). Doxorubicin attenuates serotonin-induced long-term synaptic facilitation by phosphorylation of p38 mitogen-activated protein kinase. J Neurosci.

[21] Moruno-Manchon JF, Uzor NE, Kesler SR (2016). TFEB ameliorates the impairment of the autophagy-lysosome pathway in neurons induced by doxorubicin. Aging.

[22] Gaman AM, Uzoni A, Popa-Wagner A (2016). The role of oxidative stress in etiopathogenesis of chemotherapy induced cognitive impairment (CICI)-“chemobrain”. Aging Dis.

[23] Gong S, Feng Y, Zeng Y (2021). Gut microbiota accelerates cisplatin-induced acute liver injury associated with robust inflammation and oxidative stress in mice. J Transl Med.

[24] Soffietti R, Trevisan E, Rudà R (2014). Neurologic complications of chemotherapy and other newer and experimental approaches. Handb Clin Neurol.

[25] The Cancer Drug Manual® http://www.bccancer.bc.ca/health-professionals/clinical-resources/cancer-drug-manual/drug-index#d-content.

[26] Myers JS (2013). Cancer- and chemotherapy-related cognitive changes: the patient experience. Semin Oncol Nurs.

[27] Wefel JS, Vardy J, Ahles T (2011). International Cognition and Cancer Task Force recommendations to harmonise studies of cognitive function in patients with cancer. Lancet Oncol.

[28] Benedict RHB (1998). Hopkins Verbal Learning Test—revised: normative data and analysis of inter-form and test-retest reliability. Clin Neuropsychol.

[29] Reitan RM (1992). (Tucson: Reitan Neuropsychology Laboratory, Length). Trail Making Test Manual for Administration and Scoring.

[30] Benton AL, Hamsher KDS (1998). (Iowa City: AJA Associates). Multilingual Aphasia Examination.

[31] Stuss DT, Stethem LL, Poirier CA (1987). Comparison of the three tests of attention and rapid information processing across six age groups. Clin Neuropsychol.

[32] Wechsler D (1997). (San Antonio, TX: The Psychological Corporation). Wechsler Adult Intelligence Scale III.

[33] Gronwall D (1997). Paced auditory serial addition task: a measure of recovery from concussion. Percept Mot Skills.

[34] Schretlen D (1989). (Lutz, FLA: Psychological Assessment Resources). The Brief Test of Attention.

[35] Nasreddine ZS, Phillips NA, Bédirian V (2005). The montreal cognitive assessment, MoCA: a brief screening tool for mild cognitive impairment. J Am Geriatr Soc.

[36] Lange M, Joly F (2017). How to identify and manage cognitive dysfunction after breast cancer treatment. J Oncol Pract.

[37] Costa DSJ, Loh V, Birney DP (2018). The structure of the FACT-Cog v3 in cancer patients, students, and older adults. J Pain Symptom Manage.

[38] Dyk KV, Crespi CM, Petersen L (2019). Identifying cancer-related cognitive impairment using the FACT-Cog perceived cognitive impairment. JNCI Cancer Spectr.

[39] Lange M, Joly F, Vardy J (2019). Cancer-related cognitive impairment: an update on state of the art, detection, and management strategies in cancer survivors. Ann Oncol.

[40] Li M, Caeyenberghs K (2018). Longitudinal assessment of chemotherapy-induced changes in brain and cognitive functioning: a systematic review. Neurosci Biobehav Rev.

[41] Simó M, Rifà-Ros X, Rodriguez-Fornells A (2013). Chemobrain: a systematic review of structural and functional neuroimaging studies. Neurosci Biobehav Rev.

[42] Kesler S, Janelsins M, Koovakkattu D (2013). Reduced hippocampal volume and verbal memory performance associated with interleukin-6 and tumor necrosis factor-alpha levels in chemotherapy-treated breast cancer survivors. Brain Behav Immun.

[43] Cheung YT, Ng T, Shwe M (2015). Association of proinflammatory cytokines and chemotherapy-associated cognitive impairment in breast cancer patients: a multi-centered, prospective, cohort study. Ann Oncol.

[44] Carroll JE, Nakamura ZM, Small BJ (2023). Elevated C-reactive protein and subsequent patient-reported cognitive problems in older breast cancer survivors: the thinking and living with cancer study. J Clin Oncol.

[45] Sorokin J, Saboury B, Ahn JA (2014). Adverse functional effects of chemotherapy on whole-brain metabolism: a PET/CT quantitative analysis of FDG metabolic pattern of the “chemo-brain”. Clin Nucl Med.

[46] Ahles TA, Saykin AJ, Noll WW (2003). The relationship of APOE genotype to neuropsychological performance in long-term cancer survivors treated with standard dose chemotherapy. Psycho-Oncology.

[47] Ng T, Teo SM, Yeo HL (2016). Brain-derived neurotrophic factor genetic polymorphism (rs6265) is protective against chemotherapy-associated cognitive impairment in patients with early-stage breast cancer. Neuro-Oncology.

[48] Small BJ, Rawson KS, Walsh E (2011). Catechol-O-methyltransferase genotype modulates cancer treatment-related cognitive deficits in breast cancer survivors. Cancer.

[49] Cheng H, Li W, Gan C (2016). The COMT (rs165599) gene polymorphism contributes to chemotherapy-induced cognitive impairment in breast cancer patients. Am J Transl Res.

[50] Sheinerman KS, Tsivinsky VG, Crawford F (2012). Plasma microRNA biomarkers for detection of mild cognitive impairment. Aging.

[51] Xie B, Zhou H, Zhang R (2015). Serum miR-206 and miR-132 as potential circulating biomarkers for mild cognitive impairment. JAD.

[52] Kenny A, McArdle H, Calero M (2019). Elevated plasma microRNA-206 levels predict cognitive decline and progression to dementia from mild cognitive impairment. Biomolecules.

[53] Koh YQ, Tan CJ, Toh YL (2020). Role of exosomes in cancer-related cognitive impairment. IJMS.

[54] Silva YP, Bernardi A, Frozza RL (2020). The role of short-chain fatty acids from gut microbiota in gut-brain communication. Front Endocrinol.

[55] Usmani MT, Krattli RP, El-Khatib SM (2023). BDNF augmentation using riluzole reverses doxorubicin-induced decline in cognitive function and neurogenesis. Neurotherapeutics.

[56] Karschnia P, Parsons MW, Dietrich J (2019). Pharmacologic management of cognitive impairment induced by cancer therapy. Lancet Oncol.

[57] Yang Y, Von Ah D (2024). Cancer-related cognitive impairment: updates to treatment, the need for more evidence, and impact on quality of life—a narrative review. Ann Palliat Med.

[58] Bernstein LJ, McCreath GA, Nyhof-Young J (2018). A brief psychoeducational intervention improves memory contentment in breast cancer survivors with cognitive concerns: results of a single-arm prospective study. Support Care Cancer.

[59] Richard NM, Bernstein LJ, Mason WP (2019). Cognitive rehabilitation for executive dysfunction in brain tumor patients: a pilot randomized controlled trial. J Neurooncol.

[60] Fardell JE, Vardy J, Shah JD (2012). Cognitive impairments caused by oxaliplatin and 5-fluorouracil chemotherapy are ameliorated by physical activity. Psychopharmacology.

[61] Winocur G, Wojtowicz JM, Huang J (2014). Physical exercise prevents suppression of hippocampal neurogenesis and reduces cognitive impairment in chemotherapy-treated rats. Psychopharmacology.

[62] Campbell KL, Zadravec K, Bland KA (2020). The effect of exercise on cancer-related cognitive impairment and applications for physical therapy: systematic review of randomized controlled trials. Phys Ther.

[63] Melis M, Schroyen G, Leenaerts N (2023). The impact of mindfulness on cancer-related cognitive impairment in breast cancer survivors with cognitive complaints. Cancer.

[64] Johns SA, Von Ah D, Brown LF (2016). Randomized controlled pilot trial of mindfulness-based stress reduction for breast and colorectal cancer survivors: effects on cancer-related cognitive impairment. J Cancer Surviv Res Pract.

[65] Huberty AR, Bernstein LJ, Sabiston CM (2023). Feasibility of a remotely-delivered yoga intervention on cognitive function in breast cancer survivors: a mixed-methods study. Front Hum Neurosci.

[66] James DL, Maxfield M, Han S (2024). Cognitive function, mood and sleep changes in response to a Tai Chi/Qigong intervention among older breast cancer survivors: an exploratory analysis. Front Cognit.

[67] Alvarez J, Meyer FL, Granoff DL (2013). The effect of EEG biofeedback on reducing postcancer cognitive impairment. Integr Cancer Ther.

[68] Liou KT, Ahles TA, Garland SN (2019). The relationship between insomnia and cognitive impairment in breast cancer survivors. JNCI Cancer Spectr.

[69] Ancoli-Israel S, Palmer BW, Cooke JR (2008). Cognitive effects of treating obstructive sleep apnea in Alzheimer's disease: a randomized controlled study. J Am Geriatr Soc.

[70] Palmer ACS, Zortea M, Souza A (2020). Clinical impact of melatonin on breast cancer patients undergoing chemotherapy; effects on cognition, sleep, and depressive symptoms: a randomized, double-blind, placebo-controlled trial. PLoS One.

[71] Giordano G, Martin-Willett R, Gibson LP (2023). Cannabis use in cancer patients: acute and sustained associations with pain, cognition, and quality of life. Explor Med.

[72] Gondi V, Pugh SL, Tome WA (2014). Preservation of memory with conformal avoidance of the hippocampal neural stem-cell compartment during whole-brain radiotherapy for brain metastases (RTOG 0933): a phase II multi-institutional trial. J Clin Oncol.

[73] Schimmel WCM, Gehring K, Eekers DBP (2018). Cognitive effects of stereotactic radiosurgery in adult patients with brain metastases: a systematic review. Adv Radiat Oncol.

[74] Brown PD, Pugh S, Laack NN (2013). Memantine for the prevention of cognitive dysfunction in patients receiving whole-brain radiotherapy: a randomized, double-blind, placebo-controlled trial. Neuro Oncol.

[75] Brown PD, Gondi V, Pugh S (2020). Hippocampal avoidance during whole-brain radiotherapy plus memantine for patients with brain metastases: phase III trial NRG oncology CC001. J Clin Oncol.

[76] Ng T, Cheung YT, Ng QS (2014). Vascular endothelial growth factor inhibitors and cognitive impairment: evidence and controversies. Expert Opin Drug Saf.

[77] Joly F, Heutte N, Duclos B (2016). Prospective evaluation of the impact of antiangiogenic treatment on cognitive functions in metastatic renal cancer. Eur Urol Focus.

[78] Mulder SF, Bertens D, Desar IM (2014). Impairment of cognitive functioning during sunitinib or sorafenib treatment in cancer patients: a cross-sectional study. BMC Cancer.

[79] Shaw AT, Bauer TM, Marinis F (2020). First-line lorlatinib or crizotinib in advanced ALK-positive lung cancer. N Engl J Med.

[80] Duong SL, Barbiero FJ, Nowak RJ (2021). Neurotoxicities associated with immune checkpoint inhibitor therapy. J Neurooncol.

[81] Rogiers A, Leys C, De Cremer J (2020). Health-related quality of life, emotional burden, and neurocognitive function in the first generation of metastatic melanoma survivors treated with pembrolizumab: a longitudinal pilot study. Support Care Cancer.

[82] Rogiers A, Leys C, Lauwyck J (2020). Neurocognitive function, psychosocial outcome, and health-related quality of life of the first-generation metastatic melanoma survivors treated with ipilimumab. J Immunol Res.

[83] Belin C, Devic P, Ayrignac X (2020). Description of neurotoxicity in a series of patients treated with CAR T-cell therapy. Sci Rep.

[84] Ruark J, Mullane E, Cleary N (2020). Patient-reported neuropsychiatric outcomes of long-term survivors after chimeric antigen receptor T cell therapy. Biol Blood Marrow Transplant.

[85] Gust J, Hay KA, Hanafi LA (2017). Endothelial activation and blood-brain barrier disruption in neurotoxicity after adoptive immunotherapy with CD19 CAR-T cells. Cancer Discov.

[86] Pan Z, Park C, Brietzke E (2019). Cognitive impairment in major depressive disorder. CNS Spectr.

[87] Park JH, Jung YS, Jung YM (2019). The role of depression in the relationship between cognitive decline and quality of life among breast cancer patients. Support Care Cancer.

[88] Mayo SJ, Edelstein K, Atenafu EG (2024). Cognitive symptoms across diverse cancers. JAMA Network Open.

[89] Jia J, Wei C, Liang J (2016). The effects of DL-3-n-butylphthalide in patients with vascular cognitive impairment without dementia caused by subcortical ischemic small vessel disease: a multicentre, randomized, double-blind, placebo-controlled trial. Alzheimer’s Dement.

[90] Jansen CE, Cooper BA, Dodd MJ (2011). A prospective longitudinal study of chemotherapy-induced cognitive changes in breast cancer patients. Support Care Cancer.

[91] Buchanan ND, Dasari S, Rodriguez JL (2015). Post-treatment neurocognition and psychosocial care among breast cancer survivors. Am J Prev Med.

[92] Lawrence JA, Griffin (2016). A study of Donepezil in female breast cancer survivors with self-reported cognitive dysfunction 1 to 5 years following adjuvant chemotherapy. J Cancer Surviv.

